# Associations of age and sex with characteristics of extracellular vesicles and protein‐enriched fractions of blood plasma

**DOI:** 10.1111/acel.14356

**Published:** 2024-10-07

**Authors:** Yiyao Huang, Junjie Feng, Jiannan Xu, Liang Dong, Wanting Su, Bo Li, Kenneth W. Witwer, Lei Zheng

**Affiliations:** ^1^ Department of Laboratory Medicine Nanfang Hospital, Southern Medical University Guangzhou Guangdong China; ^2^ Department of Molecular and Comparative Pathobiology Johns Hopkins University School of Medicine Baltimore Maryland USA; ^3^ Department of Urology Renji Hospital, Shanghai Jiao Tong University School of Medicine Shanghai China; ^4^ Department of Neurology Johns Hopkins University School of Medicine Baltimore Maryland USA; ^5^ Richman Family Precision Medicine Center of Excellence in Alzheimer's Disease Johns Hopkins University School of Medicine Baltimore Maryland USA

**Keywords:** age, biomarker, extracellular vesicles, plasma, proteins, sex

## Abstract

Extracellular vesicles (EVs) are nanosized particles that are released by various cell types and play vital roles in intercellular communication. They carry biological molecules reflecting the physiological and pathological states of their source cells and tissues, showing potential as biomarkers. However, the impact of demographic factors like age and sex on the properties of blood plasma EVs remains underexplored. This study aims to fill this gap by evaluating how these factors influence the particle count and proteomic profiles of plasma EV preparations and corresponding protein fractions. Plasma samples from 120 healthy volunteers were collected and pooled into six groups: young males (age: 27.6 ± 4.0), young females (27.4 ± 3.8), middle‐aged males (48.8 ± 3.8), middle‐aged females (48.9 ± 3.9), old males (69.3 ± 3.9), and old females (69.4 ± 4.3). EV‐ and protein‐enriched fractions were separated by size‐exclusion chromatography (SEC). Fractions were characterized for particle number concentration and protein composition to identify characteristics affected by age and biological sex. Plasma EVs and corresponding protein fractions exhibited distinct characteristics, with differential enrichment of markers related to EVs and other blood components, including lipoproteins. Proteomic profiles of both EVs and protein fractions displayed sex‐ and age‐dependent differences. Differentially abundant proteins displayed functions previously identified in the context of aging and sex differences, highlighting their utility as biomarkers. Age and sex significantly affect the characteristics of plasma EVs and proteins, potentially influencing their efficacy and interpretation as biomarkers in clinical applications. This study lays the groundwork for detailed mechanistic research to understand how EVs mediate age‐ and sex‐related effects in health.

## Graphical abstract

This study reveals that age and sex significantly influence the proteomic profiles of plasma extracellular vesicles (EVs) and associated protein fractions, revealing distinct protein abundance differences linked to demographic factors. These findings underscore the impact of age and sex on the interpretation of EVs as biomarkers and provide a foundation for further mechanistic research into how EVs contribute to age‐ and sex‐related variations in health.

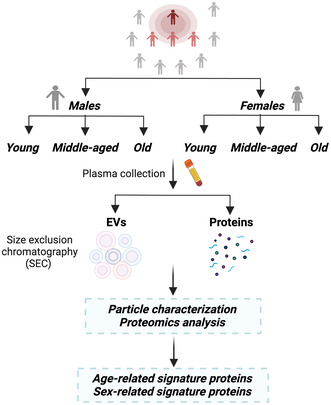

**Abbreviations**
ALB: Albumin
APO: Apolipoprotein
DE: Differential expression
EVs: Extracellular vesicles
F: Female
Hb: Hemoglobin
HDL: High‐density lipoprotein
LDL: Low‐density lipoprotein
LPs: Lipoproteins
M: Male
Mid: Middle‐aged
NFCM: Nano‐flow cytometry measurement
NTA: Nanoparticle tracking analysis
NVEPs: Non‐vesicular extracellular particles
PDP: Platelet‐depleted plasma
PROT: Proteins
RBC: Red blood cell
SEC: Size‐exclusion chromatography
TEM: Transmission electron microscopy
VLDL: Very‐low‐density lipoprotein

## 1 INTRODUCTION

Extracellular vesicles (EVs), nanosized lipid bilayer‐delimited particles released by various cell types, play a crucial role in intercellular communication (Théry et al., 2018; Welsh et al., 2024). EVs transport biological molecules—including proteins, nucleic acids, and lipids—that reflect the physiological and pathological states of their originating cells. As they are easily detectable in biofluids (Clayton et al., 2019; Erdbrugger et al., 2021; Ogawa et al., 2008) such as blood, urine, and saliva, EVs are promising candidates as minimally invasive biomarkers. EVs are recognized for their potential in the early detection, prognosis, and monitoring of diseases (Huang et al., 2022; Huang, Liao, et al., 2023; Lee, Ni, et al., 2023; Leggio et al., 2021; Wang et al., 2021) including cancer, neurodegenerative disorders, cardiovascular diseases, and infections.

Separating EVs from blood plasma presents several challenges (Dong et al., 2020; Ter‐Ovanesyan et al., 2023; Vergauwen et al., 2021) due to the small size and heterogeneity of EVs, as well as the complex mixture of non‐EV components in plasma. Plasma is rich in free proteins, protein aggregates, and non‐vesicular extracellular particles (NVEPs), which can co‐isolate with EVs because of overlapping physical characteristics, leading to potential misinterpretation of results (Simonsen, 2017; Welsh et al., 2024). NVEPs such as lipoproteins (LPs), which transport lipids, are particularly problematic. The size and density overlap between EVs and LPs results in co‐isolation of LPs with EVs (Piontek & Roos, 2022), leading to the potential presence of non‐EV structural lipids alongside EVs. This is particularly an issue in studies employing precipitation‐based methods, which tend to precipitate more non‐EV protein aggregates (Karttunen et al., 2019; Lobb et al., 2015). Despite these challenges, there is a notable lack of studies that directly compare EVs with non‐EV protein components in plasma, especially in the context of biomarker discovery.

Moreover, while there is extensive research on the impact of various pathological conditions on EV contents (Sun et al., 2022; Yin et al., 2021), studies examining the impact of patient baseline characteristics—such as age and biological sex at birth—on plasma EV properties are lacking (Noren Hooten et al., 2022). Considering the association of many diseases with age and sex (“2021 Alzheimer's disease facts and figures,” 2021; Colafella & Denton, 2018; Hoit et al., 2022), a thorough understanding of these demographic influences on EVs under physiology conditions is crucial. However, the few existing studies on aging and EVs mostly focus on specific molecular targets rather than comprehensive profiling (Alberro et al., 2019; Baek et al., 2016; Eitan et al., 2017; Enjeti et al., 2017; Lazo et al., 2021; Zhang et al., 2020), limiting our understanding of the full scope of age‐related changes. Similarly, research on the impact of sex on EV composition is scarce, especially in normal physiology (Baek et al., 2016; Enjeti et al., 2017; Gustafson et al., 2015; Noren Hooten et al., 2019). In addition, the use of precipitation‐based methods for plasma EV enrichment in several studies (Eitan et al., 2017; Lazo et al., 2021; Noren Hooten et al., 2019; Zhang et al., 2020) making it challenging to differentiate the contributions of EVs and co‐isolated proteins.

To address these gaps, this study focused on how age and sex influence the proteome of plasma EVs and protein fractions, using a well‐designed cohort of healthy donors. We used size‐exclusion chromatography (SEC) to separate larger EV‐enriched fractions (EVs) and smaller protein‐enriched fractions (PROT) from blood plasma (Boing et al., 2014; Huang, Liao, et al., 2023). Detailed subsequent analysis confirmed the distinct proteome of EVs and PROT, revealing differential enrichment of markers related to EVs and other blood components, including apolipoproteins specifically. Our findings demonstrate sex‐ and age‐dependent variations in the proteomic profiles of both EVs and PROT across young, middle‐aged, and old donors. These results revealed the effects of age and sex on plasma EVs and PROT, underlining the importance of considering baseline characteristics in biomarker research and development.

## 2 METHODS

### 2.1 Clinical plasma collection

Whole blood samples collected at the Department of Laboratory Medicine, Nanfang Hospital, Southern Medical University, Guangzhou, China in a study approved by the Ethics Committee of Nanfang Hospital, Southern Medical University, and all donors provided informed consent (Approval registration number: NFBC‐2022‐003). Samples were collected from healthy, overnight‐fasted donors until 120 samples that satisfied inclusion criteria had been obtained. Specifically, the 120 samples were from 40 donors in each of three age categories (young, middle‐aged, and old), with 20 males and females in each age group. Details of age and biological sex distribution are found in Table 1.

**TABLE 1:** Clinical plasma samples used in this study.

Groups	Abbreviation	Age (average ± SD)	Age (range)	HDL (mg/dl)	LDL (mg/dl)	ALB (g/L)	Number before pooling	Number after pooling
Young‐ male	Young‐M	27.6 ± 4.0	22–34	46.5 ± 7.3	110.7 ± 22.9	45.1 ± 2.4	20	5
Young‐female	Young‐F	27.4 ± 3.8	22–35	58.7 ± 12.4	95 ± 18.6	43.6 ± 4.0	20	5
Middle‐aged‐male	Mid‐M	48.8 ± 3.8	43–55	43.8 ± 9.0	130.9 ± 22.8	44.7 ± 2.1	20	5
Middle‐aged‐female	Mid‐F	48.9 ± 3.9	41–54	63.6 ± 12.1	126.6 ± 33.1	43.8 ± 2.0	20	5
Old‐ male	Old‐M	69.3 ± 3.9	64–79	52.55 ± 12.9	117.1 ± 40.0	43.6 ± 2.3	20	5
Old‐ female	Old‐F	69.4 ± 4.3	64–76	60.9 ± 13.6	126.9 ± 29.0	42.5 ± 1.7	20	5

From each donor, 5 mL whole blood was collected into an EDTA tube using a vacuum blood collection system with a 22 G needle. The procedure was conducted in accordance with the “Guidelines of Venous Blood Specimen Collection (WS/T 661‐2020)” issued by the National Health Commission. Within 2 h after blood collection, sequential centrifugation steps were used to remove blood cells (1500 x *g*, 5 min) and platelets (two times 2500 x *g*, 15 min each). In the resulting platelet‐depleted plasma (PDP), levels of albumin (ALB), very‐low‐density lipoprotein (VLDL), LDL, and HDL were measured by Beckman AU5831 Clinical Chemistry Analyzer. Lipemia and hemolysis were assessed by visual inspection and samples that exhibited color differences when compared to a hemolytic chart or that showed turbidity were discarded. From the final 120 PDP samples, pools were prepared using 500 μL PDP from each of four unique donors (i.e., six groups, five pools per group, and four donors per pool). To ensure comparability, a balanced age distribution was maintained between the pools (see details in Table S1).

### 2.2 EV‐ and protein‐enriched fraction separation

EV‐ and protein (PROT)‐enriched fractions were separated from PDP as previously published (Huang, Liao, et al., 2023). In brief, 2 mL pooled PDP were loaded onto qEV10/70 nm SEC columns (Izon Science) and eluted with PBS in 2‐ml fractions. After eluting a void buffer volume of 14.1 mL, EV (fractions (F) 1–4) and PROT (F9‐12) fractions were separately pooled and concentrated from 8 mL to 0.6 mL using 100 kDa MWCO concentrators (Thermo Fisher, 88,524). All fractions were stored at −80°C.

### 2.3 Nanoparticle tracking analysis (NTA)

Particle number of EV preparations was measured in scatter mode (488 nm laser) with a ZetaView Particle Tracking Analyzer and ZetaView software version 8.04.02 (Particle Metrix, Germany). 2 μL EV samples were diluted to 1 mL and injected into the viewing chamber by syringe. Light scattering was recorded for 2 min with the following settings: Focus: autofocus; Camera sensitivity for all samples: 70.0; Shutter: 70; Cell temperature: 25°C. Analysis parameters were: Maximum particle size 1000; Minimum particle size 5; Minimum particle brightness 30.

### 2.4 Nano‐flow cytometry measurement (NFCM)

Particle number of EV preparations was assessed by nano‐flow (NFCM, Flow NanoAnalyzer, NanoFCM) as described previously (Huang, Arab, et al., 2023). 2 μL sample was diluted from 1:100 to 1:500 in DPBS and measured for 1 min by side‐scatter calibrated for concentration with 200 nm silica beads (NanoFCM).

### 2.5 Transmission electron microscopy (TEM)

Samples (10 μL) were adsorbed to copper mesh grids at room temperature for 1 min. Followed by sterile distilled water wash, grids absorbed with samples were stained in 1% uranyl acetate solution for 1 min. After being dried under incandescent light, grids were immediately observed and photographed with a transmission electron microscope (Hitachi Ltd., H‐7650).

### 2.6 Data‐independent acquisition tandem mass spectrometry

250 μL of EV and PROT (*n* = 3 per group, as listed in Table S1) were subjected to ultrasonic lysis for protein extraction and quantitated by BCA protein assay (Pierce™ BCA Protein Assay Kit, Thermo Fisher). 10 μg of extracted proteins from each sample was then subjected to a simple and integrated spintip‐based technology (SISPROT) (SISPRO kits, BayOmics) (Chen et al., 2016; Xue et al., 2018) for protein reduction, alkylation, digestion, and desalting, with spintips packed sequentially with C18 membrane and cation exchange beads. Peptides from each sample were processed using the UltiMate 3000 liquid chromatography system (Thermo Fisher Scientific), interfaced with the timsTOF Pro 2 ion‐mobility spectrometry quadrupole time‐of‐flight mass spectrometer (Bruker Daltonics). 200 ng final peptides were reconstituted in 0.1% formic acid (FA) and separated by HPLC (AUR3‐15075C18, IonOpticks) with a 60‐min gradient. The column flow rate was maintained at 400 nL/min with column temperature at 50°C. Data‐independent acquisition (DIA) was conducted using the diaPASEF (parallel accumulation‐serial fragmentation) mode. 22 precursor isolation windows, each 40 units of mass‐to‐charge ratio (m/z) wide and covering a m/z range from 349 to 1229, were defined, allowing for comprehensive peptide capture and analysis. To synchronize with the MS1 cycle time and maximize data acquisition efficiency, variable repetition steps (ranging from 2 to 5) were implemented within the 13‐scan diaPASEF sequence during experimental runs. During PASEF MS/MS scanning phase, a linearly ramped collision energy, tailored to the ion mobility of the analytes, ranging from 59 eV (at 1/K0 = 1.6Vs/cm^2^) to 20 eV (at 1/K0 = 0.6 Vs/cm^2^), was used.

### 2.7 Mass spectrometry data processing and analysis

DIA raw data were processed and analyzed by Spectronaut 17 (Biognosys AG, Switzerland) with default settings. MS/MS spectra were searched against *Homo_sapiens*_SP (20,361, 20,220,317) from NCBI. Trypsin specificity was used for digestion. Carbamidomethyl on cysteine was set as the fixed modification. Methionine oxidation was specified as the variable modification. Dynamic iRT was used for precise retention time prediction. Spectronaut automated data extraction was guided by extensive mass calibration, adjusting the extraction windows based on internal retention time calibration and chromatographic gradient stability. The false discovery rate (FDR) cutoffs were set at 1% for both precursor and protein levels. Decoy sequences were set using a mutated strategy to apply a random number of amino acid swamps (min = 2, max = length/2). Data normalization was set to Local normalization, and protein quantification was performed using the MaxLFQ algorithm, applying only to peptides meeting the 1% FDR threshold.

To identify differentially abundant proteins between EVs and PROT, ratios of protein abundance in EVs and PROT from the same pool were calculated. Proteins exhibiting a ratio >1 or <1 in all pools (*n* = 18) were defined as differentially expressed (DE). Additionally, to explore age‐ and sex‐related differences, the ratios of protein levels between all pools from two comparison group were calculated, with three pools per group, resulting in nine ratios. Proteins consistently showing a ratio >1 or <1 between two groups were categorized as DE. This analysis includes age group comparisons within each sex (young versus middle‐aged, young versus old, and middle‐aged versus old) and sex comparisons within each age group (males versus females) separately.

Protein interaction, protein function, and tissue enrichment predictions were done by Protein–Protein Interaction Networks Functional Enrichment Analysis (STRING). Venn diagrams and volcano plots were generated in R. Hierarchical clustering was conducted by Heatmapper (Babicki et al., 2016).

### 2.8 Data and methods availability

Statistical significance of differences in raw plasma protein levels and EV particle concentration from different groups were assessed by Fisher's Least Significant Difference (LSD) test. Proteomics data files are available on request. We have submitted all relevant data of our experiments to the EV‐TRACK knowledgebase (Van Deun et al., 2017) (EV‐TRACK ID: EV240039).

## 3 RESULTS

### 3.1 Plasma, EV‐ and protein‐ enriched fraction characterization

**FIGURE 1:**
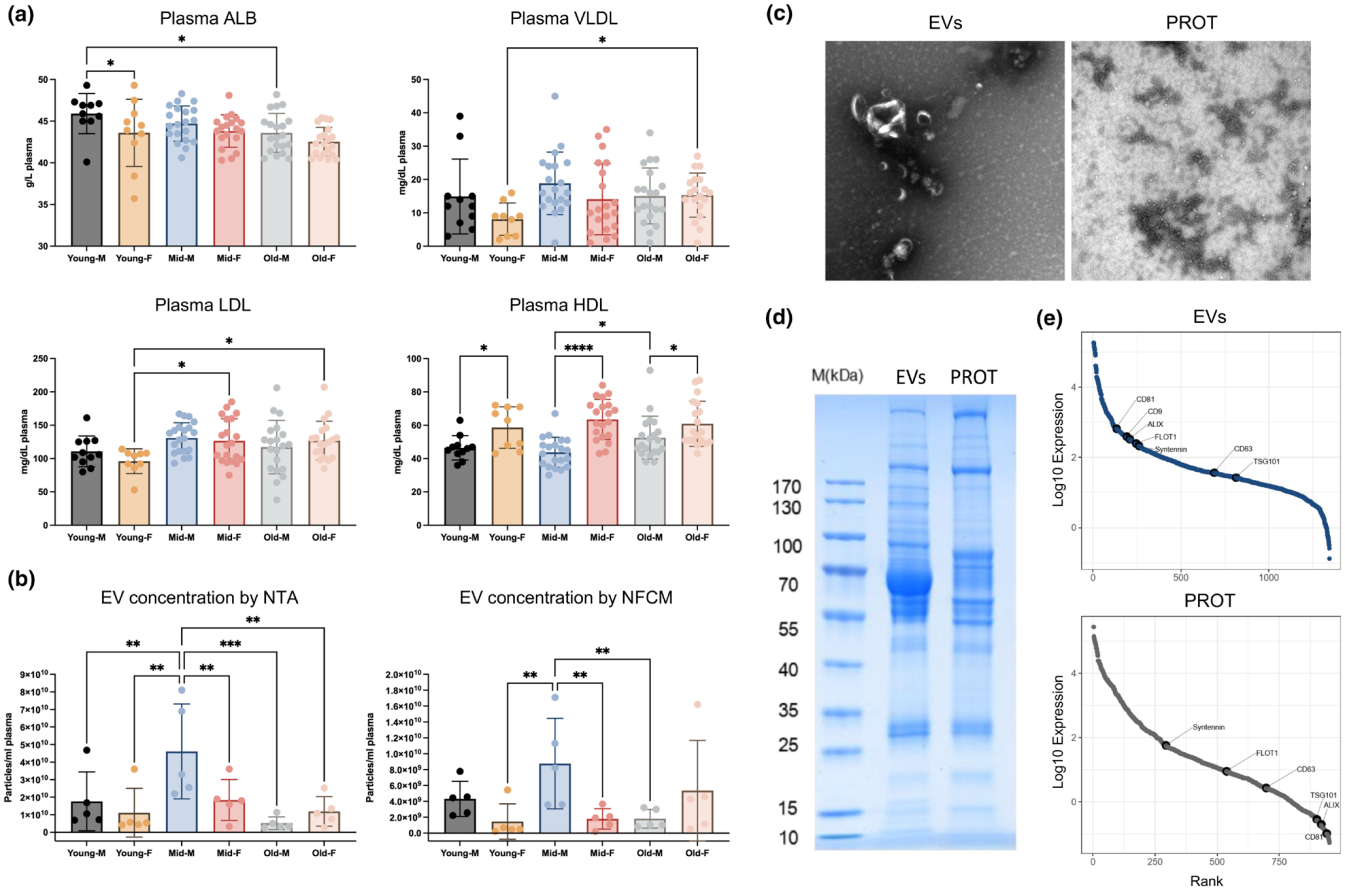
Characterization of whole plasma and EV‐ and protein‐enriched fractions. (a) Levels of albumin (ALB), very‐low‐density lipoprotein (VLDL), low‐density lipoprotein (LDL), and high‐density lipoprotein (HDL) in plasma samples. The concentration of ALB was presented as gram (g)/Liter (L), and the levels of VLDL, LDL, and HDL as milligram (mg)/Deciliter (dL). (b) Particle concentrations of EV preparations from different groups were measured by NTA (left) and NFCM (right). Particle concentration for each group was normalized by particles per milliliter (ml) of plasma. (c) Extracellular vesicle‐enriched (EVs) and protein‐enriched (PROT) fractions were visualized by negative staining transmission electron microscopy (TEM). TEM is representative of five images taken of each sample. (d) Equivalent protein from EVs and PROT were separated by gel electrophoresis and visualized with Coomassie Blue. (e) The log 10 normalized expression level and rank of typical EV markers in EVs and PROT detected by proteomics. (a, b) data are presented as mean ± SD. ns, no significant difference (*p*  ≥  0.05), *p < 0.05, **p ≤ 0.01, ***p ≤ 0.001, ****p ≤ 0.0001 by Fisher's Least Significant Difference (LSD) test.

Before plasma pooling and EV separation, components in plasma that may affect EV separation or co‐isolated with EVs were tested in raw plasma from six age/sex‐defined groups (males/females x young, middle‐aged, old; Figure 1a). The levels of ALB, VLDL, and LDL were mostly consistent across groups, with a few labelled differences. However, plasma HDL levels were higher in females compared with males in all three age groups.

SEC‐separated EVs were characterized by particle count, phenotyping, and proteomics, while PROT were analyzed for phenotyping and proteomics. Particle concentration of EVs, tested by both NTA and NFCM, revealed higher EV recovery from middle‐aged males compared with other groups (Figure 1b). Transmission electron microscopy (TEM) showed round to oval particles with characteristic EV morphology in EVs, but not obvious in PROT (Figure 1c). Gel electrophoresis revealed distinct total protein distribution between EVs and PROT (Figure 1d). By mass spectrometry proteomics, EV‐related proteins, including CD81, CD9, CD63, FLOT1, Syntenin, and TSG101, ranked high among all tested proteins in EVs but were either lower or undetected in PROT (Figure 1e).

### 3.2 Proteome characteristics in EV‐ and protein‐enriched fractions

**FIGURE 2:**
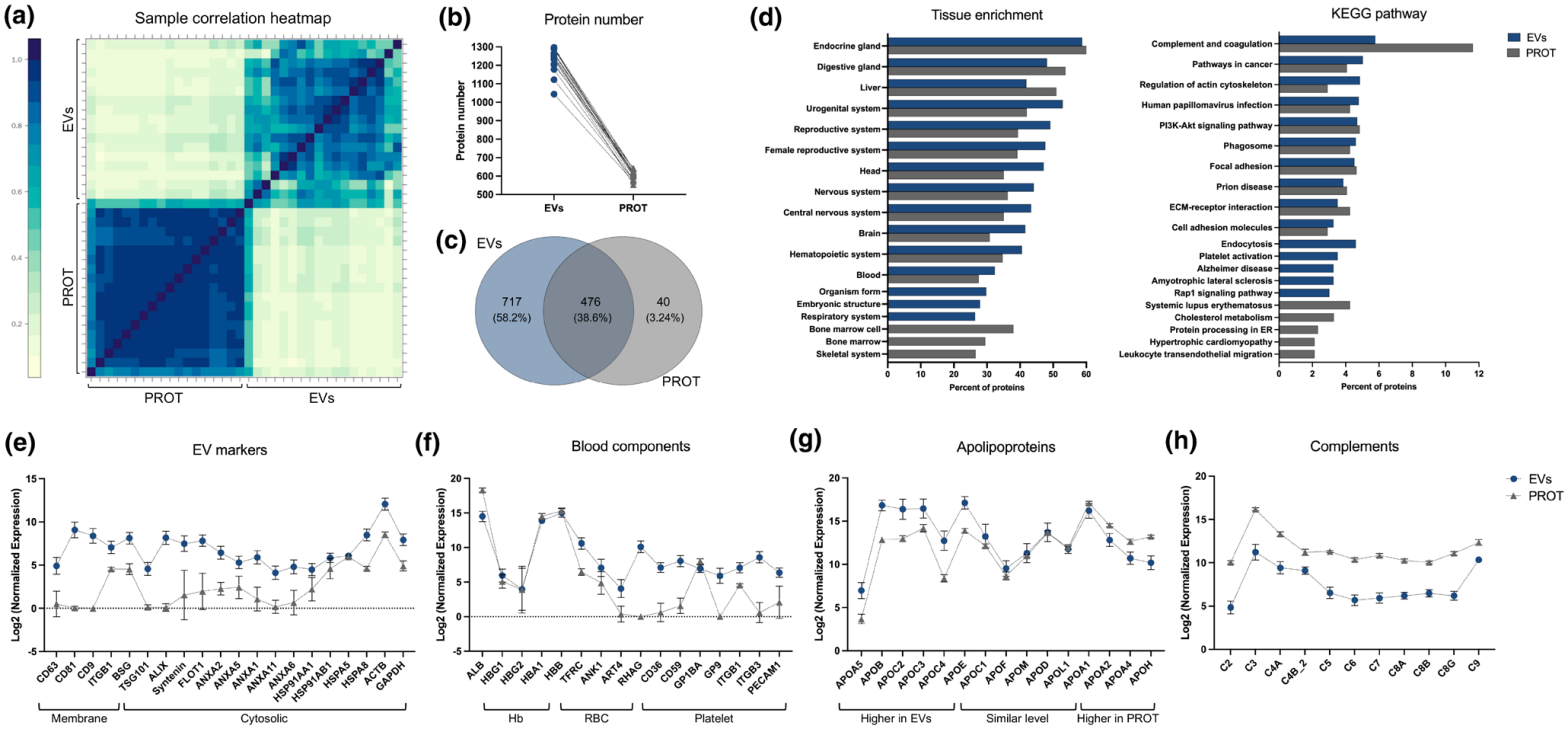
Proteomics of EVs and PROT. (a) Correlation matrix showing Pearson correlation coefficients for normalized protein expression between EVs and PROT. (b) Number of unique proteins identified in EVs and PROT. (c) Venn diagrams of all proteins identified in EVs and PROT. (d) Top 15 tissue enrichment and KEGG pathways ranked by identified protein number in EVs and PROT by the Protein–Protein Interaction Networks Functional Enrichment Analysis (STRING). (e–h) The log 2 normalized expression level of EV markers (e), blood component markers (f), apolipoproteins (g), and complements (h) in EVs and PROT. Data are presented as mean ± SD.

Correlation of the overall proteome of EVs and PROT was examined across all pools. The relative similarities among pools within the EV and PROT groups and distinctions between EVs and PROT are shown in Figure 2a. Additionally, levels of individual proteins in EVs and PROT were assessed by Pearson's correlation analysis (Figure S1a). The results demonstrated a significant correlation of average protein levels in EVs and PROT (*R* = 0.502, *p* < 0.01). The number of proteins identified in EVs and PROT are shown in Figure 2b and Table S2. Notably, EVs exhibited a higher protein diversity compared with paired PROT. For subsequent analyses, proteins detected in more than 80% of pools in EV and PROT groups were included. Among the 1193 proteins in EVs and 516 in PROT, 476 proteins (38.6%) were common to both groups, 717 proteins (58.2%) were exclusively detected in EVs, and only 40 proteins (3.2%) were exclusive to PROT (Figure 2c).

Distinct tissue and KEGG pathway enrichment patterns of proteins detected in EV and PROT were revealed by STRING analysis. Among the top 15 tissue‐related terms ranked by the number of identified proteins, common terms, including endocrine gland, digestive gland, and liver, showed higher representation in PROT than in EVs. Conversely, common terms related to various body systems, including urogenital, reproductive, nervous, and hematopoietic systems were higher in EVs compared with PROT. In addition, certain terms were exclusively enriched in a single fraction, such as embryonic structure and respiratory system in EVs and bone marrow and skeletal system in PROT (Figure 2d, left panel). For KEGG enrichment analysis, these proteins showed enrichment in both common and distinct pathways in EVs and PROT. Shared pathways include complement and coagulation, cancer, infectious diseases, cell integration, and adhesion. In contrast, EVs were enriched in pathways related to endocytosis and neurodegenerative diseases, while PROT were enriched in inflammatory and cholesterol metabolism pathways (Figure 2d, right panel).

### 3.3 EVs versus PROT: Putative EV, blood component, lipoprotein markers, and complements

Based on literature, established database, and MISEV suggestions (Théry et al., 2018; Welsh et al., 2024), we examined known protein markers associated with EVs (Figure 2e) and other blood components (Figure 2f), including apolipoproteins specifically (Figure 2g). Seventy‐two proteins in EVs and 31 in PROT were among the top 100 most common EV proteins as reported by both Vesiclepedia (Chitti et al., 2024) and Exocarta (Keerthikumar et al., 2016) (Figure S1b, Table 2). All of these proteins were more abundant in EVs than in PROT, except for gelsolin (GSN) and alpha‐2‐macroglobulin (A2M) (Figure S1c). Additionally, 20 EV markers as mentioned in MISEV2018 were also highly enriched in EVs compared with PROT, including both tetraspanins and cytosolic proteins (Figure 2e). Among them, CD63, CD81, CD9, TSG101, ALIX, and ANXA11 were absent in most PROT.

**TABLE 2:** Protein list of EV and protein enriched fractions matched to top 100 markers in Exocarta and Vesiclepedia databases (*n* = 72).

Detected in EVs and PROT	Only in EVs
A2M	CD63
ACTB	CD81
ALDOA	CD9
ANXA2	ALIX
ANXA5	Syntennin
CFL1	FLOT1
CLTC	ACLY
EEF2	ACTN4
ENO1	AHCY
FLNA	ANXA1
GAPDH	ANXA11
GSN	ANXA6
HSP90AA1	ATP1A1
HSP90AB1	BSG
HSPA5	CCT2
HSPA8	CCT3
ITGB1	CCT5
LDHA	CDC42
LDHB	CLIC1
LGALS3BP	EZR
PFN1	GDI2
PGK1	GNAI2
PKM	GNAS
PPIA	GNB1
PRDX1	GNB2
PRDX2	KPNB1
RAN	MFGE8
SLC3A2	MSN
TFRC	RAB5C
TPI1	RAB7A
YWHAB	RAC1
	RAP1B
	RHOA
	TCP1
	TSG101
	UBA1
	VCP
	YWHAE
	YWHAG
	YWHAQ
	YWHAZ

Highly abundant blood proteins include albumin (ALB), proteins related to hemoglobin (Hb), red blood cells (RBCs), and platelets, and apolipoproteins (APOs). ALB was highly enriched in PROT versus EVs (Figure 2f). In contrast, Hb proteins were similarly abundant in EVs and PROT, while RBC and platelet proteins were mostly enriched in EVs versus PROT, except for ankyrin‐1 (ANK1) and platelet glycoprotein Ib alpha chain (GP1BA) (Figure 2f). Among apolipoproteins (APOs), APOA5, B, C2, C3, C4, and E were more abundant in EVs. Conversely, APOA2, A4, and H were more abundant in PROT than in EVs. Additionally, the levels of some APOs, including APOA1, F, and M, which are commonly associated with lipoproteins (LPs), and APO D and L1, which are less associated with LPs, had similar levels in EVs and PROT (Figure 2g). Furthermore, upon examining common but differentially abundant proteins in EVs and PROT (listed in Table S3), complement components were found to be more abundant in PROT than their paired EVs (Figure 2h).

### 3.4 Age‐associated proteins in EVs and PROT: Males versus females

**FIGURE 3:**
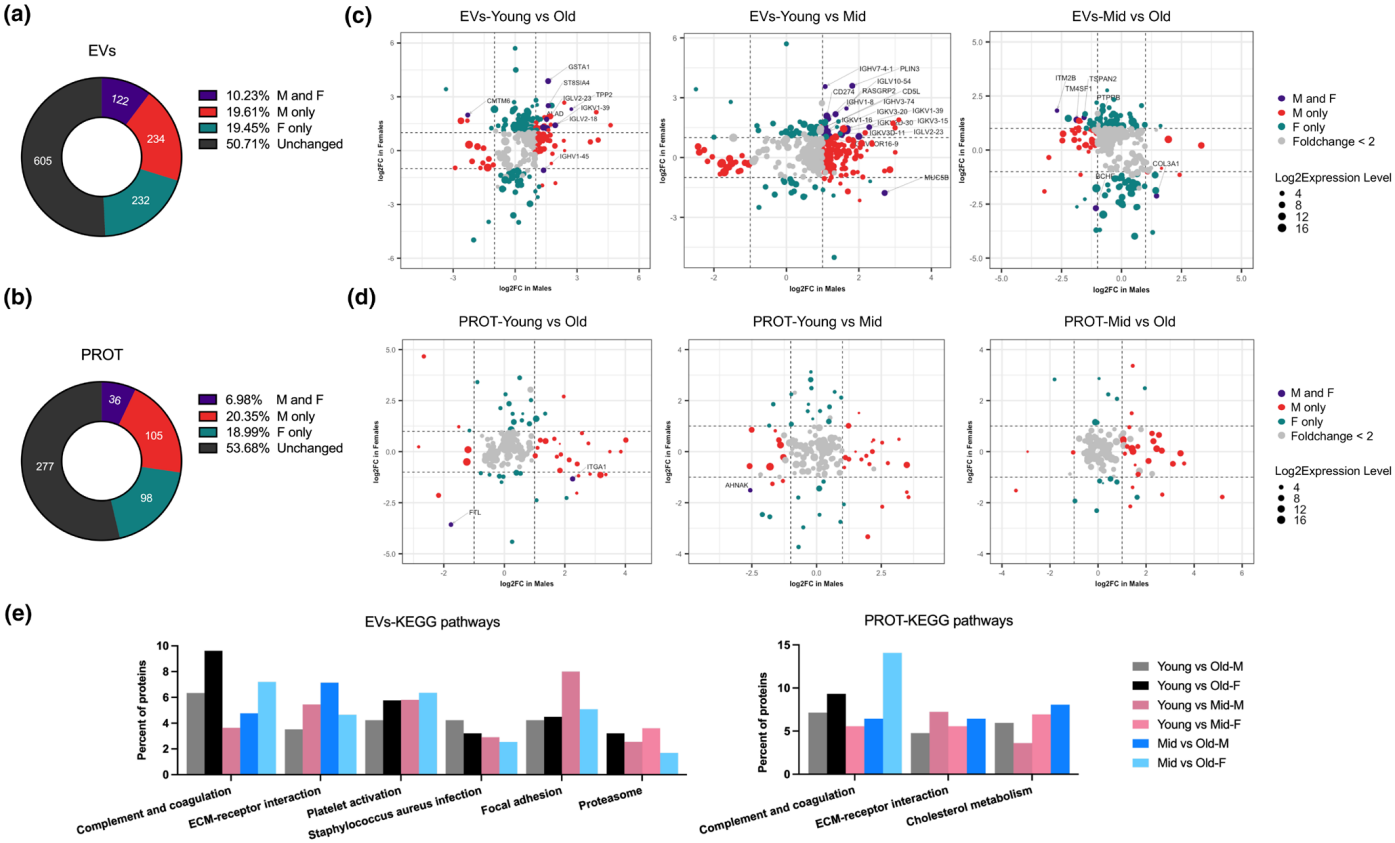
Age‐associated protein differences in males and females. (a, b) Numbers of proteins in EVs and PROT with differential expression (DE) across comparisons (young versus old, young versus middle‐aged (mid), or mid versus old) in males (M) and females (F). (c, d) Log 2 fold change (Log2FC) of DE proteins of young versus old (left), young versus mid (middle), and mid versus old (right) in M versus F for EVs (c) and PROT (d). Dashed lines indicate log2FC of one (up or down). Colored dots indicate DE proteins significantly changed in M only (red), in F only (green), or in both sexes (purple), or unchanged proteins (grey). Dot size represents the mean Log 2 normalized expression levels of individual proteins. (e) Common KEGG pathways identified in at least four of six comparisons across age and sex by the Protein–Protein Interaction Networks Functional Enrichment Analysis (STRING).

To identify proteins associated with age, comparisons were made between young versus middle‐aged (mid), young versus old, and mid versus old groups for males (M) and females (F), separately (Table 3). 50.71% (Figure 3a) and 53.78% (Figure 3b) of the proteins detected in EVs and PROT were consistently abundant across age groups. One hundred and twenty‐two proteins (10.23%) in EVs and 36 proteins (6.98%) in PROT had differences in at least one of the comparisons in both M and F. Additionally, sex‐dependent changes were observed, with proteins altered exclusively in either M or F in EVs and PROT (Figure 3a,b).

The log_2_ fold change (Log_2_FC) of differentially expressed (DE) proteins in M and F were compared for EVs (Figure 3c) and PROT (Figure 3d). In EVs, most DE proteins with fold change >2 between different age groups were also exclusively detected in either M or F, while a small number of proteins exhibited differences in both sexes (Figure 3c, highlighted by purple dots with the protein names indicated). The levels of these highlighted proteins were mostly higher in young versus old (Figure 3c, left panel) or mid groups (Figure 3c, middle panel). In PROT, only three proteins with fold change >2 were differentially expressed in both sexes (as labelled in Figure 3d).

KEGG pathway enrichment analysis was performed on DE proteins identified from six comparative analyses: young versus old, young versus mid, mid versus old in both M and F, separately. Pathways commonly enriched in at least four of these comparisons for EVs and PROT are shown in Figure 3e. Notably, pathways of complement and coagulation, along with ECM‐receptor interaction, were consistently enriched among DE proteins from both EVs and PROT across several comparisons.

**TABLE 3:** The number of differentially expressed proteins (DEP) in EVs and PROT between age groups.

Fractions	Sex	Age comparisons	DEP number	Up	Down
EVs	M	Young versus. Old	142	30	112
		Young versus Mid	275	26	249
		Mid versus Old	88	62	26
	F	Young versus Old	156	40	116
		Young versus Mid	111	34	77
		Mid versus Old	236	63	173
PROT	M	Young versus Old	84	27	57
		Young versus Mid	83	32	51
		Mid versus Old	62	23	39
	F	Young versus Old	75	28	47
		Young versus Mid	72	32	40
		Mid versus Old	71	33	38

### 3.5 Dynamic protein changes across age groups: Comparisons of three groups

**FIGURE 4:**
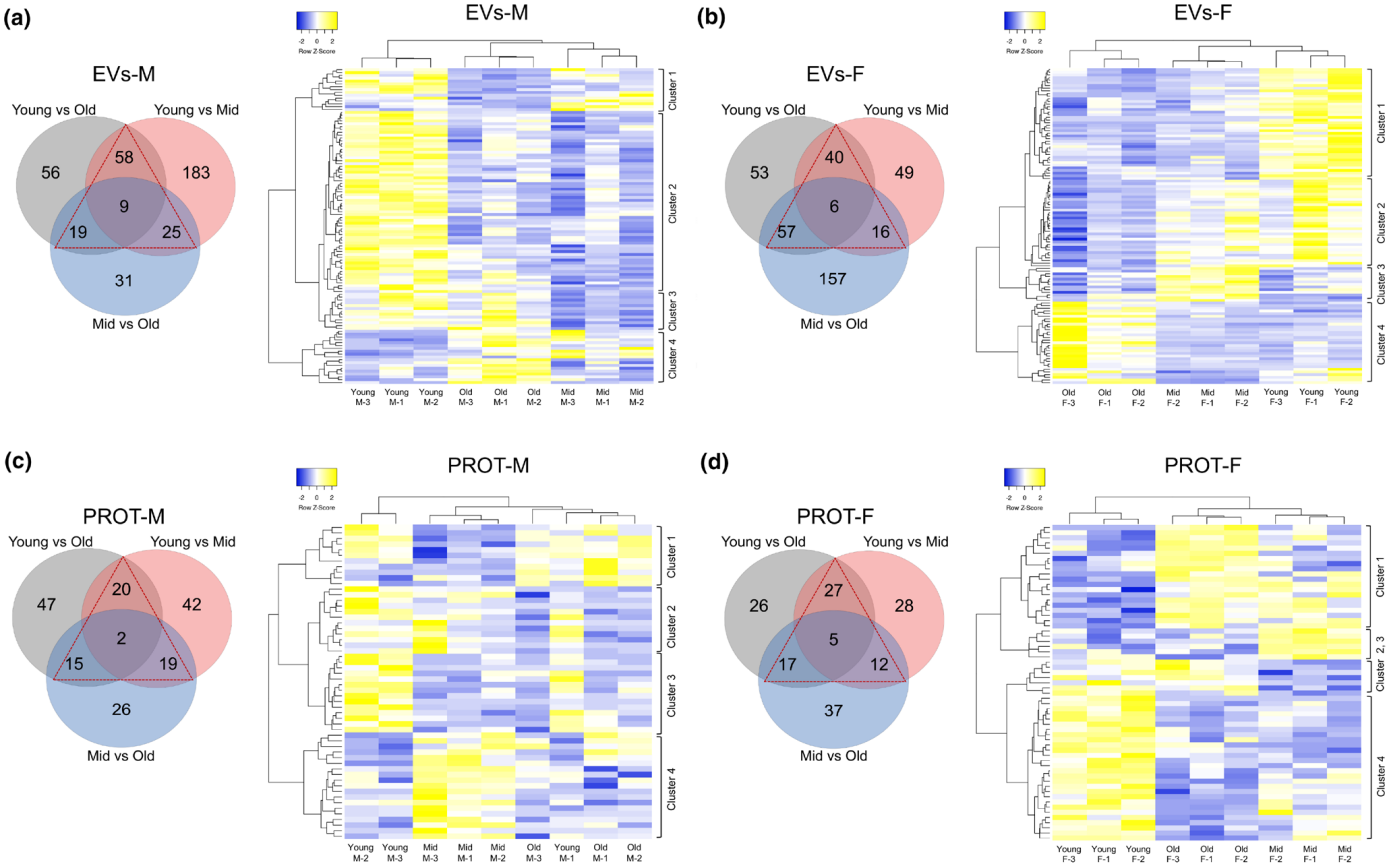
Protein clusters associated with age in males and females. Venn diagrams (left panels) of differential expression (DE) proteins in EVs from males (M) (a) and females (F) (b), and in PROT from M (c) and F (d) across age group comparisons: Young versus old, young versus mid, and mid versus old. Proteins that are DE in at least two out of three age comparisons are marked with triangles. The heatmaps (right panels) depict unsupervised hierarchical clustering of young, mid, and old pools based on the normalized expression of these marked proteins, shown for EVs from M (a) and F (b), and PROT from M (c) and F (d).

To identify proteins exhibiting dynamic changes, differentially expressed (DE) proteins among three age groups in EVs and PROT were compared (Figure 4a–d). Only a small number of proteins displayed differences in all three comparisons in EVs‐M (*n* = 9), EVs‐F (*n* = 6), PROT‐M (*n* = 2), and PROT‐F (*n* = 5). Upon assessing the levels of these overlapping proteins, individual proteins that progressively changed with age in EVs and PROT were identified (Figure S2a,b).

We further analyzed proteins displaying alterations in more than two comparisons (highlighted with red triangles) using unsupervised hierarchical clustering. In EVs‐M (Figure 4a), EVs‐F (Figure 4b), and PROT‐F (Figure 4d), the young, mid, and old groups clustered together separately based on these DE proteins, whereas this distinct clustering pattern was not observed in PROT‐M (Figure 4c). Overall, the heatmap analysis revealed that these proteins exhibited different expression patterns in specific comparisons. For example, proteins in cluster 1 of EVs‐M (Figure 4a) were less abundant in the old group compared with both the young and mid groups, while cluster 2 proteins were higher abundant in the young group compared with both the old and mid groups. Protein clusters (1–4) with distinct expression patterns between groups are labeled on the heatmaps (Figure 4a–d).

**FIGURE 5:**
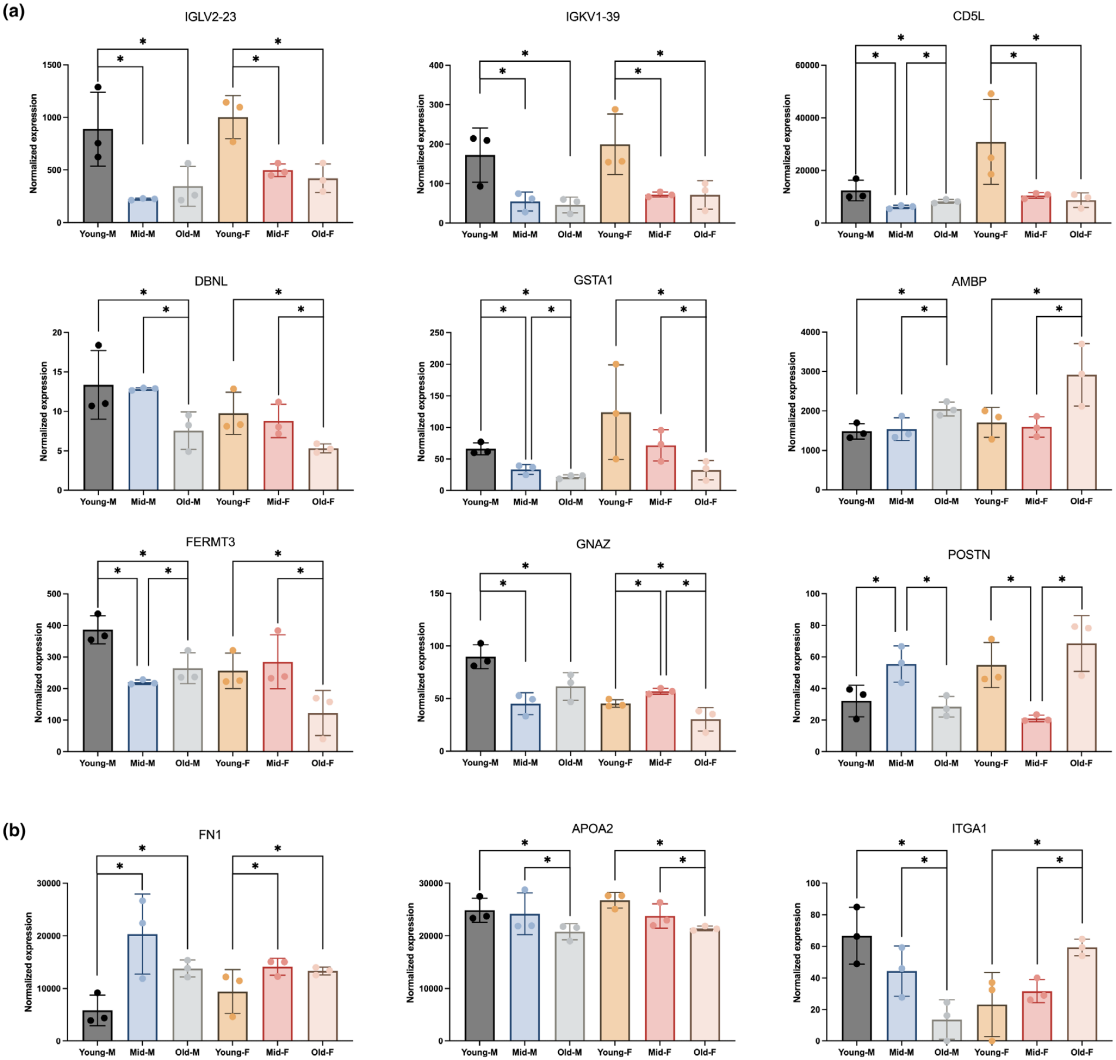
Age‐associated proteins common in males and females. Proteins that were differentially expressed (DE) across biological sex and exhibited alterations in more than two of the age group comparisons. The intersecting proteins are displayed separately for EVs (a) and PROT (b).

To identify sex‐common proteins that dynamically changed with age, we selected DE proteins that were consistent across sexes, and then cross‐referenced these proteins with those exhibiting alterations in more than two of the age group comparisons. The proteins found to overlap in these analyses are shown for EVs (Figure 5a) and PROT (Figure 5b).

### 3.6 Sex‐associated proteins in EVs and PROT across age groups

**FIGURE 6:**
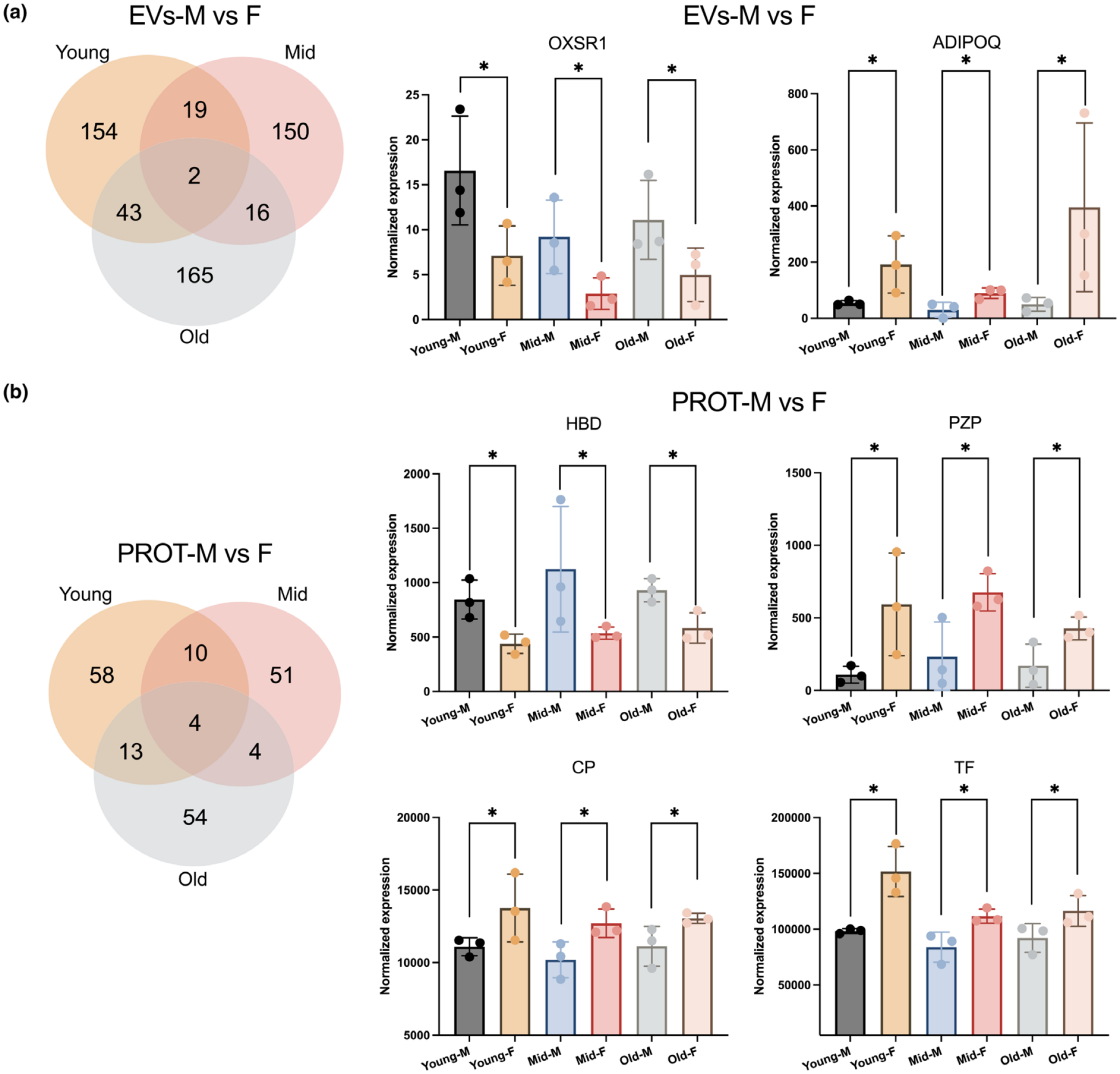
Sex‐associated protein levels across different age groups. (a, b) Venn diagrams (left panels) of proteins showing differential expression (DE) between M and F in EVs (a) and PROT (b) of young, mid, and old groups. Normalized expression of proteins differing between males and females across all three age groups (right panels) in EVs (a) and PROT (b).

Since many age‐related proteins are also sex‐dependent, proteins with differential abundance between M and F were also examined within the young, mid, and old groups separately. While numerous proteins exhibited differences between M and F in each age group (Figure 6a,b, left panels), only two proteins in EVs (Figure 6a) and four in PROT (Figure 6b) were consistently different between M and F across all three age groups. DE proteins that differed in more than two age groups were visualized by unsupervised hierarchical clustering (Figure S3a,b). While most pools within M and F clustered together based on the levels of these DE proteins, variances were observed between pools.

## 4 DISCUSSION

Exploration of the effects of baseline patient characteristics on the composition and properties of blood plasma EVs and PROT under physiological conditions remains limited. We sought to address this gap in the context of age and sex, using SEC‐ separated EVs and PROT. Our results reveal fraction‐dependent particle characteristics and protein contents but also highlight sex‐ and age‐dependent proteomic differences. Since donor demographic parameters may be associated with outcomes of plasma component measurements, we recommend that future studies incorporate detailed patient information into biomarker discovery and development, including data analysis.

To explore the potential effects of blood proteins on EV separation, we for the first time revealed the distinct yet correlated proteome of SEC separated EV‐ and PROT‐enriched fractions. SEC is commonly used to separate EVs from plasma, exploiting their size disparity from individual proteins and small NVEPs, including lipoproteins (LPs) (Boing et al., 2014; Dong et al., 2020; Gamez‐Valero et al., 2016; Huang, Liao, et al., 2023). Our findings indicate that SEC‐separated EVs exhibit a greater diversity of proteins compared with PROT, including a significant enrichment of typical EV markers. In contrast, ALB, often seen as a contaminant in EV separation (Pietrowska et al., 2019), is markedly less abundant in EVs. However, some protein retention between EVs and PROT post‐SEC was also evident. For instance, some highly abundant blood proteins, such as APOs that are more or less associated with lipids and LPs, were similarly abundant in EVs and PROT. This could be explained by SEC incompletely separating EVs from LPs with the overlapped size, but also by interactions of EVs, APOs, lipids, and LPs, for example, through the formation of protein coronas on the surfaces of EVs (Toth et al., 2021; Wolf et al., 2022) or direct LP‐EV interactions (Busatto et al., 2022; Sodar et al., 2016).

The proteomic distinctions between EV‐ and PROT‐enriched fractions may allow researchers to select the most information‐rich fractions for disease/organ‐specific studies. While PROT fractions show stronger associations with glandular systems, such as endocrine and digestive, EVs demonstrate broader systemic connections. Specifically, EVs are enriched with biomarkers related to neurodegenerative diseases, highlighting their relevance in these disorders. Additionally, PROT fractions contain higher levels and a more extensive enrichment of complement system pathways, indicating a potentially more significant role in complement function. These differences in tissue enrichment support previous findings (Oh et al., 2023) that blood‐based biomarkers can provide insights into organ‐specific health and disease conditions. Moreover, more proteins in EVs are influenced by age and sex compared with PROT, suggesting that EVs could serve as more effective biomarkers for changes related to these factors. Conversely, the lesser variability in PROT may offer advantages for baseline normalization in research studies.

Our study uncovered molecules associated with age in the blood of males and females, including numerous age‐related proteins that are sex‐specific. These proteomic signatures may be influenced by differences in the regulation of endocrine, immune, and cognitive systems across sexes (Fischer & Riddle, 2018; Thomas et al., 2019). However, some shared function enrichments were observed in these proteins, implicating common age‐related biological pathways such as the complement and coagulation cascades, ECM‐receptor interactions, and platelet activation (Armento et al., 2021; Michaud et al., 2013; Wang et al., 2019). These findings align with previous research on how age impacts immune system‐related proteins in plasma (Oh et al., 2023). Furthermore, our findings indicate the presence of a small subset of age‐associated proteins that are common to both sexes, representing potential universal biomarkers for age. These proteins are implicated in immune responses and inflammatory processes, aligning with established research that links these physiological changes with aging (Borgoni et al., 2021; Franceschi et al., 2018; Oh et al., 2023; Shuken et al., 2022; Walker et al., 2022).

We also revealed hallmark proteins associated with sex in both EVs and PROT across young, middle‐aged, and old groups, underscoring their potential as biomarkers for sex‐based differences. In EVs, females exhibited lower oxidative stress responsive kinase 1 (OXSR1) levels, aligning with lower oxidative stress markers typically found in female blood samples (Tower et al., 2020). Adiponectin (ADIPOQ) levels were higher in females, reflecting recognized differences in fat metabolism and associated diseases between the sexes (Ohman‐Hanson et al., 2016; Yannakoulia et al., 2003). In PROT, elevated levels of estrogen‐inducible serum protein (Ilies et al., 2017) and pregnancy zone protein (PZP) in females further highlighted the influence of sex hormones on protein expression in the bloodstream. In addition, the distinct profiles of sex‐associated proteins found in three age groups likely reflect hormonal and metabolic shifts that accompany aging (Faulkner & Belin de Chantemele, 2019; Lee, Richard, et al., 2023; Olivieri et al., 2023).

Our study sheds light on the largely unexplored effects of age and sex on EVs and blood proteins, identifying promising targets for future mechanistic and biomarker discovery research. Specifically, we identified age‐ and sex‐related proteins in both larger EV‐ and smaller protein‐enriched (PROT) blood fractions, suggesting that these blood‐based signatures could serve as biomarkers for aging and provide insights into the roles of EVs in mediating age‐ and sex‐related effects. By focusing on the tissue enrichment distinctions between EVs and PROT, researchers can select the most relevant fractions for disease‐ or organ‐specific investigations.

Notably, our work emphasizes the importance of considering various factors, such as age, sex, EV separation techniques, and presence of co‐isolated components in all stages of EV studies, from designing experiments to analyzing and interpreting results. While our study primarily focuses on protein components, we acknowledge that non‐protein components of plasma, such as nucleic acids and lipids, are also crucial mediators. Future research into these components, particularly in relation to age and sex, could further our understanding of plasma biomarker development. Additionally, examining the organ‐specific origins of age‐related EVs could provide valuable insights into the complexities of aging across tissues and organs, potentially leading to new diagnostic or therapeutic approaches.

## AUTHOR CONTRIBUTIONS

Conceptualization was undertaken by LZ and KWW. Clinical samples were collected by YH and WS. Experiments and data analysis were collaboratively conducted by YH, JF, JX, LD, WS, and BL. Supervision of the project was provided by LZ and KWW. The original draft of the manuscript was written by YH, JF, and JX, while the review and editing were carried out by LZ, KWW, and YH.

## FUNDING INFORMATION

This work was supported by the National Natural Science Fund for Distinguished Young Scholars of China (82025024) and National Key Research and Development Program of China (2021YFA1300604) to LZ, and by the National Natural Science Foundation for Young Scientists of China (Grant No. 82302637) to YH. KWW is supported in part by CA241694, AI144997, MH118164, DA047807, the Paul G. Allen Frontiers Group, and the Richman Family Precision Medicine Center of Excellence in Alzheimer's Disease.

## CONFLICT OF INTEREST STATEMENT

KWW has a sponsored research agreement with Ionis Pharmaceuticals; is or has been an advisory board member of ShiftBio, Exopharm, NeuroDex, NovaDip, and ReNeuron; holds stock options with NeuroDex; and performs ad hoc consulting as Kenneth Witwer Consulting.

## Supporting information


Figures S1–S3.

Tables S1–S3.

## Data Availability

Proteomics data files are available on request. We have submitted all relevant data of our experiments to the EV‐TRACK knowledgebase (Van Deun et al., 2017) (EV‐TRACK ID: EV240039).
