# Supplementary material for: Associations of age and sex with characteristics of extracellular vesicles and protein‐enriched fractions of blood plasma
Source: Aging Cell. 2024 Oct 7;24(1):e14356. doi: 10.1111/acel.14356 (PMC11709091; doi:10.1111/acel.14356)
Supplement: Supplementary file 1 — Figures S1–S3. [file ACEL-24-e14356-s002.zip › ACEL_14356_Sup Figure Captions.docx]

# Supplementary figure legends

**Supplementary figure 1.** **Proteomics differences in EVs and PROT.** A) Correlations of average protein levels in EVs and PROT. Linear regression lines are shown in purple. Pearson correlation coefficient (R) and significance (p) are shown based on 18 pools. B) Venn diagrams of all proteins identified in EVs and PROT, and top 100 most common EV proteins as reported by Vesiclepedia and Exocarta databases. C) Unsupervised hierarchical clustering of EVs and PROT based on the normalized expression of 31 common EV proteins.

**Supplementary figure 2. Proteins progressively changed with age in EVs and PROT.** Proteins that were differentially expressed (DE) in all age group comparisons. The protein names and levels are displayed separately for EVs-M and F (A), and PROT-M and F (B).

**Supplementary figure 3.** **Sex-associated protein clusters in EVs and PROT.** The heatmaps depict unsupervised hierarchical clustering of all pools based on the normalized expression of sex associated proteins differed in more than two age groups, shown for EVs (A) and PROT (B).
