# Supplementary figures and images for: Associations of age and sex with characteristics of extracellular vesicles and protein‐enriched fractions of blood plasma
Source: Aging Cell. 2024 Oct 7;24(1):e14356. doi: 10.1111/acel.14356 (PMC11709091; doi:10.1111/acel.14356)

Supplementary Figure-1

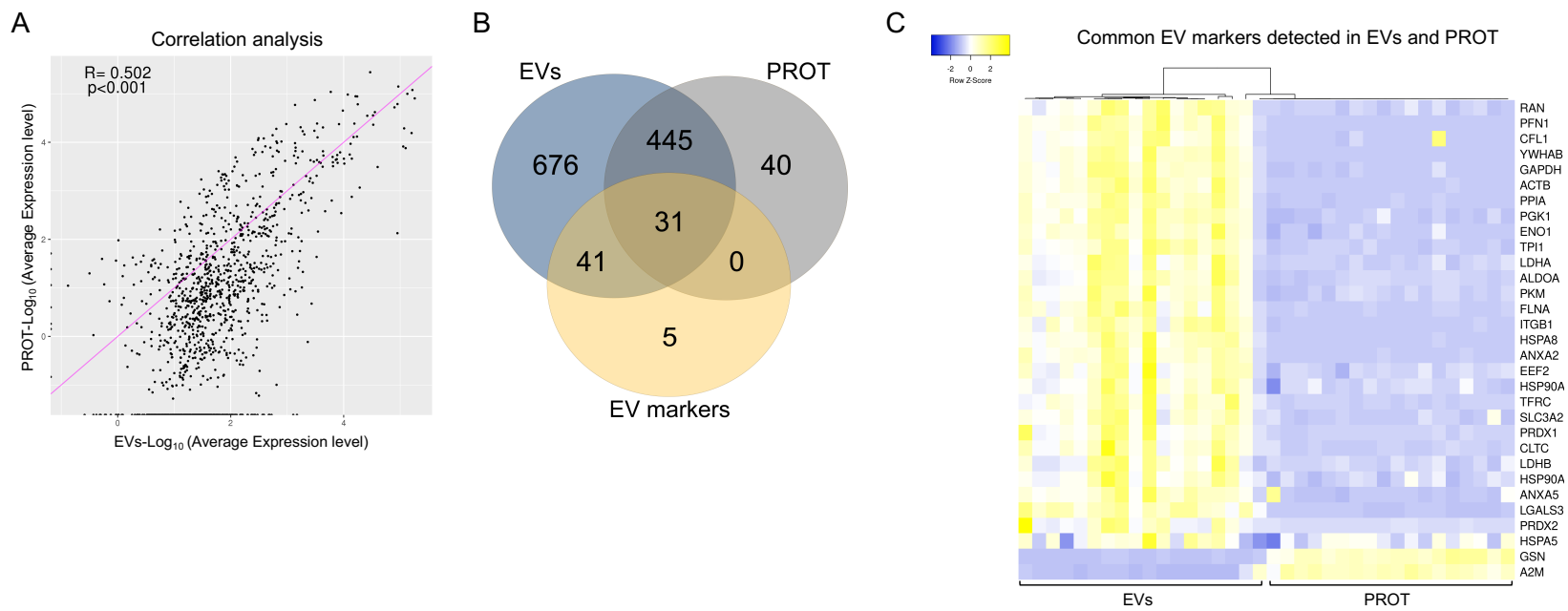

Supplementary Figure-2

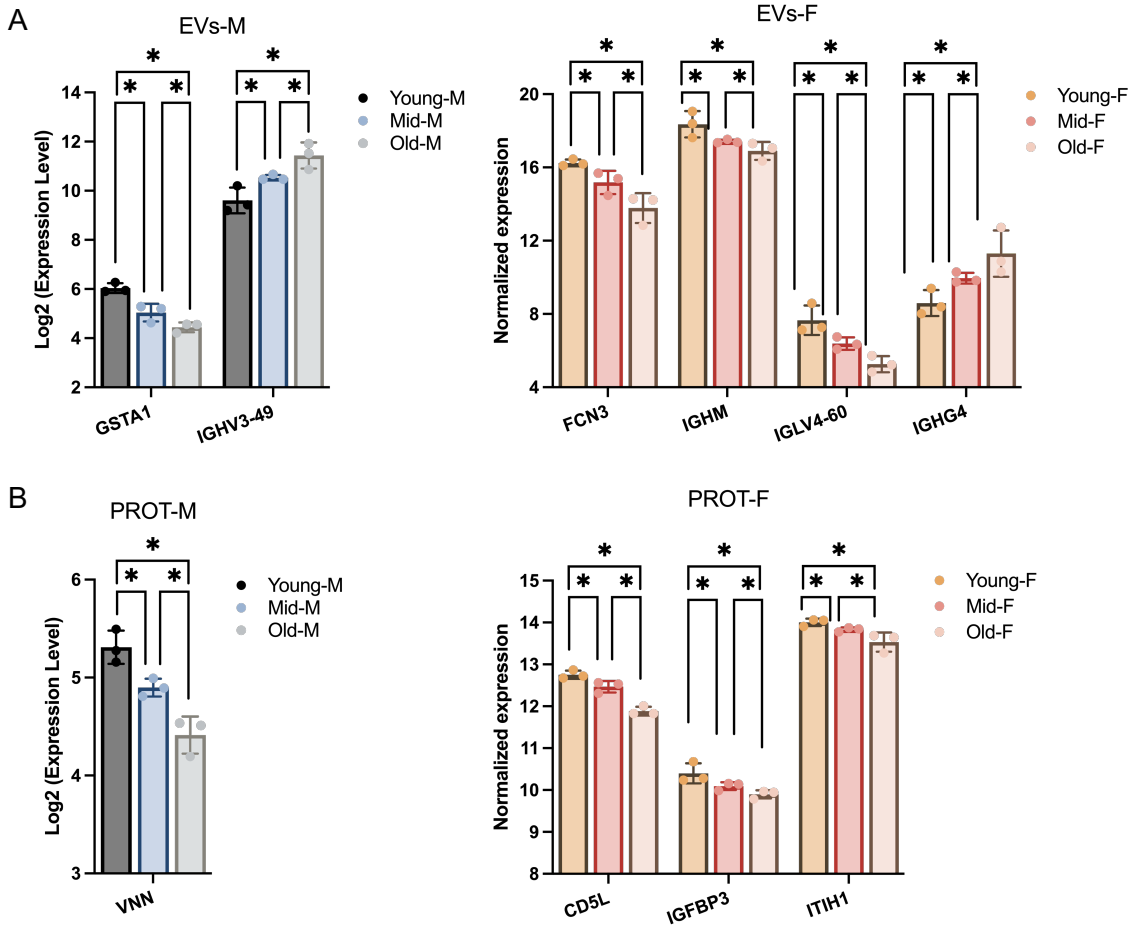

Supplementary Figure-3

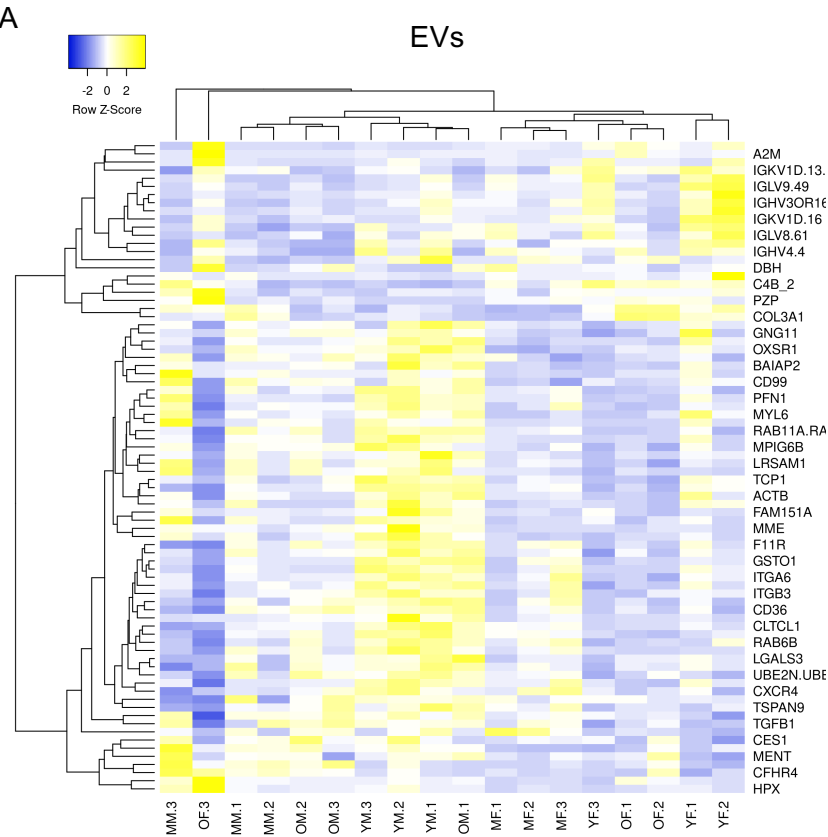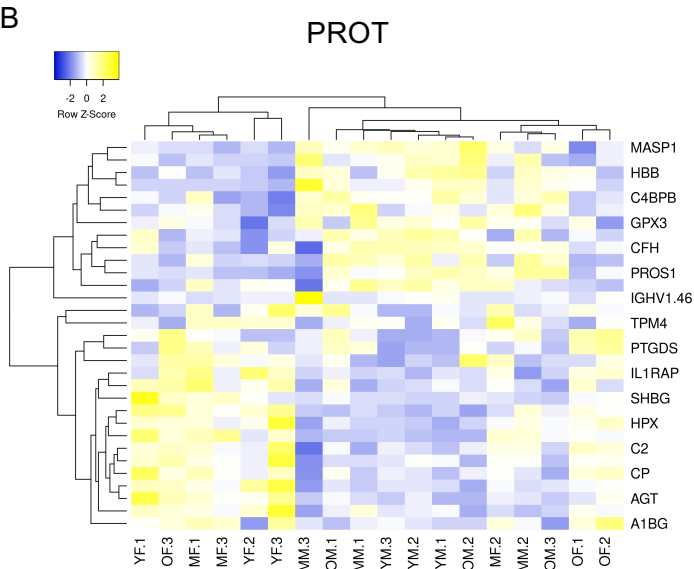

Supplement: Supplementary file 1 — Figures S1–S3. [file ACEL-24-e14356-s002.zip › Supporting information_Sup_figures.pdf]
